# Cartilage and Muscle Cell Fate and Origins during Lizard Tail Regeneration

**DOI:** 10.3389/fbioe.2017.00070

**Published:** 2017-11-02

**Authors:** Ricardo Londono, Wei Wenzhong, Bing Wang, Rocky S. Tuan, Thomas P. Lozito

**Affiliations:** ^1^Department of Orthopaedic Surgery, Center for Cellular and Molecular Engineering, University of Pittsburgh School of Medicine, Pittsburgh, PA, United States; ^2^Molecular Therapy Laboratory, University of Pittsburgh School of Medicine, Pittsburgh, PA, United States

**Keywords:** lizard, cartilage, muscle, tail, regeneration, blastema, dedifferentiation, epimorphosis

## Abstract

**Introduction:**

Human cartilage is an avascular tissue with limited capacity for repair. By contrast, certain lizards are capable of musculoskeletal tissue regeneration following tail loss throughout all stages of their lives. This extraordinary ability is the result of a complex process in which a blastema forms and gives rise to the tissues of the regenerate. Blastemal cells have been shown to originate either from dedifferentiated tissues or from existing progenitor cells in various species, but their origin has not been determined in lizards. As reptiles, lizards are the closest relatives to mammals with enhanced regenerative potential, and the origin of blastemal cells has important implications for the regenerative process. Hence, the aim of this study is to determine the cellular origin of regenerated cartilage and muscle tissues in reptiles using the mourning gecko lizard as the regenerative model.

**Methods:**

To trace the fate and differentiation potential of cartilage during tail regeneration, cartilage cells pre-labeled with the fluorescent tracer Dil were injected into lizard tails, and the contribution of cartilage cells to regenerated tail tissues was assessed by histologic examination at 7, 14, and 21 days post-tail amputation. The contribution of muscle cells to regenerated tail tissues was evaluated using muscle creatine kinase promoter-driven Cre recombinase in conjunction with the Cre-responsive green-to-red fluorescence shift construct CreStoplight. 21 days after amputation, tail tissues were analyzed by histology for red fluorescent protein (RFP)-positive cells.

**Results:**

At 7 days post-amputation, Dil-labeled cartilage cells localized to the subapical space contributing to the blastema. At 14 and 21 days post-amputation, Dil-labeled cells remained in the subapical space and colocalized with Collagen type II (Col2) staining in the cartilage tube and myosin heavy chain (MHC) staining in regenerated muscle. Lineage tracing of myocytes showed colocalization of RFP with Col2 and MHC in differentiated tissues at 21 days post-amputation.

**Conclusion:**

This study demonstrates that differentiated cartilage cells contribute to both regenerated muscle and cartilage tissues following tail loss, and in turn, differentiated muscle cells contribute to both tissue types as well. These findings suggest that dedifferentiation and/or transdifferentiation are at least partially responsible for the regenerative outcome in the mourning gecko.

## Introduction

Cartilage damage usually occurs as a result of physical trauma or degenerative disease (Aurich et al., 2014; Naraghi and White, 2016; Saxby and Lloyd, 2017) oftentimes resulting in substantial pain, loss of function, and significant health-care costs (Bhatia et al., 2013; Losina et al., 2015; Brittberg et al., 2016). Unfortunately, cartilage is an avascular tissue with very limited capacity for spontaneous repair (Hunter, 1995), and although treatment strategies are available—including microfracture, mosaicplasty, and osteochondral allografts, these options have limited effectiveness and significant failure rates (Lewis et al., 2006; Farr and Yao, 2011; Tetteh et al., 2012).

In contrast to humans, certain lizard species including scincids, gekkotans, lacertids, and anoles are capable of regenerating cartilage and other musculoskeletal tissues at all stages of life (Moffat and Bellairs, 1964; Bellairs and Bryant, 1985; Alibardi, 2010; Fisher et al., 2012). When faced with a predatorial threat, these species have the ability to undergo tail autotomy—a defense mechanism through which the lizard can shed or discard its tail to distract the predator and escape the attack (Woodland, 1920; Moffat and Bellairs, 1964), and to then regenerate the missing appendage during the weeks following the event. Although some anatomical differences exist between the original tail and its regenerated counterpart—including a different scale pattern and a modified arrangement of skeletal muscle (Kamrin and Singer, 1955; Simpson, 1964; Gilbert et al., 2015), some of these differences such as the replacement of the original vertebrae with a cartilage tube that resists ossification are particularly interesting because they indicate that cartilage regeneration is, at least in some species, mechanistically possible (Lozito and Tuan, 2015).

The extraordinary regenerative response observed in lizards—known as epimorphic regeneration (Morgan, 1901)—is the result of a complex process that begins with hemostasis and re-epithelialization of the open wound immediately after tail loss. As these processes take place, soft tissues retract into the tail stump and a thickened specialized signaling epithelium known as the apical epithelial cap (AEC) begins to form (McLean and Vickaryous, 2011). The diameter of the wound starts to decrease (Cox, 1969) and cells localized distally to the original spinal cord begin to aggregate underneath the AEC resulting in the formation of the blastema (Woodland, 1920; Werner, 1967; Bellairs and Bryant, 1985; Delorme et al., 2012). The blastema is a pool of progenitor cells that becomes apparent as early as 1 week after tail loss (McLean and Vickaryous, 2011) and has the remarkable capability to give rise to the differentiated tissues of the regenerated tail, including skeletal muscle and cartilage tissue in the distal portion of the cartilage tube (French et al., 1976; Bryant et al., 1981).

Not surprisingly, the origin of cells that contribute to the blastema and eventually become the regenerated tissues has been a topic of great interest and debate not only in lizards, but in other regenerative species as well (Slack, 2006). Originally thought to be composed of a homogenous cell population, blastemal cells are now known to represent a heterogeneous population of what appear to be lineage-restricted progenitor cells (Kragl et al., 2009). The origin of blastemal cells has been investigated in “super-healing” anamniote organisms including newts and salamanders (Kragl et al., 2009; Sandoval-Guzmán et al., 2014), but the specific source of reptilian blastemal cells remains largely unknown. Since its identification in lizards, blastemal cells have been proposed to originate either from dedifferentiated tissues that acquire the ability to differentiate into other lineages as they course through the blastemal state (Needham, 1965; Burgess, 1967; Bellairs and Bryant, 1985), or from adult progenitor cells that reside in pre-existing niches and become activated when the need arises (e.g., following autotomy) (Kahn and Simpson, 1974; Zhou et al., 2013; Alibardi, 2014).

This study aims to determine the cellular origin of the differentiated cartilage and muscular tissues in the regenerated lizard tail using the mourning gecko lizards (*Lepidodactylus lugubris*) as regenerative model. The mourning gecko is a particularly versatile organism for two reasons. First, as a parthenogenic species with chromosomal polymorphism (Volobouev and Pasteur, 1988; Trifonov et al., 2015), it allows for transplantation of cells and tissues among members of the same colony without rejection, and second, as reptiles, lizards are the only amniotes with extraordinary musculoskeletal healing abilities and therefore are the closest relatives to mammals with enhanced natural regenerative potential. The mourning gecko is one of the only species that is diploid, parthenogenetic, and capable of tail regeneration. Taken together, these features make the mourning gecko an attractive model for the study of tissue regeneration and repair (Alibardi, 2010).

## Materials and Methods

All procedures were approved by and performed according to the guidelines of the Institutional Animal Care and Use Committee at the University of Pittsburgh (Protocol Number 15114947).

### Cartilage Cell Isolation and Culture

Cartilage cells were isolated from cartilage tubes in mourning geckos and cultured *in vitro* for 2 weeks prior to transplantation (*n* = 4). Briefly, cartilage tubes were isolated using sterile technique as previously described (Lozito and Tuan, 2015) and washed three times in Leibovitz’s L-15 medium (Gibco). Cells were isolated by placing the cartilage tubes in digestion solution and incubated for 1 h at 37°C: 40 mg trypsin (Gibco), 50 mg of collagenase II (Sigma), and 40 ml of HBSS (Gibco) containing penicillin/streptomycin (Gibco). Digestion was stopped by adding 10 ml of fetal bovine serum (FBS) (Gibco). The suspension containing dissociated cells was then filtered through a 40-µm cell strainer and the cells were centrifuged at 1,500 rpm and resuspended in cartilage cell growth media: 440 ml DMEM/F12, 50 ml FBS, 5 ml 1:1:1 penicillin/streptomycin/fungizone, 5 ml Glutamax, 5% chicken embryo extract (Gemini Bioproducts), and 20 ng/ml FGF-2 (Peprotech). Cells were plated on T-75 uncoated flasks (*n* = 4) for culture at a density of approximately five tail yield/flask. Cells were cultured to confluence (2 weeks) with media changes every 3–4 days.

### Cartilage Cell Dil Labeling and Transplantation

Dil labeling of cartilage cells was performed using CellTracker™ CM-Dil (Molecular Probes, Invitrogen) following the manufacturer’s instructions. Briefly, after culturing for 2-week cells were trypsinized and incubated in suspension with 1 µM Dilute Vybrant^®^ CM-Dil labeling solution for 5 min at 37°C followed by an additional 15-min incubation at 4°C. Cells were then washed with phosphate-buffered saline (PBS) and resuspended at a density of 5,000 cells/μl. The Dil-labeled cartilage cell suspension (2.5 million cells/animal) was then injected intramuscularly in the dorsal region of the lizard tail using a BD insulin syringe and a microinjector system (Sutter Instrument). Following injection, Dil-labeled cartilage cells were allowed to engraft for 24 h and tails were amputated at injection sites. Regenerated tails were then collected at 7 days, 14 days, and 21 days post-initial amputation (*n* = 4 animals per time point).

### Myocyte Lineage Tracing

Muscle creatine kinase (MCK)-Cre plasmids were constructed by replacing CAG promoters in pCAG-Cre expression plasmids (Addgene Plasmid #13775) with tMCK promoters (Wang et al., 2008). CreStoplight constructs were acquired from Addgene (Plasmid #37402). Plasmids were purified *via* CsCl gradients and resuspended in 10 mM Tris–HCl (pH 8.5) at 1.0 µg/µl. MCK-Cre and CreStoplight plasmid solutions were mixed 1:1 (1.0 µg/µl total DNA concentration) and injected (5 µl) into lizard tail blastemas (10 days postamputation) using a microinjection system (Sutter Instrument). An ECM 830 square wave electroporation system (BTX) and a pair of paddle electrodes (BTX) were used for electroporation. Five 50-V pulses with a length of 50 m and an interval of 1 s were applied to each blastema after injection. Treated tails regenerated for 2 weeks and were re-amputated. A fluoresce dissecting microscope (Leica) were used to visualize transfected muscle bundles during tail amputations. Re-amputated tails regenerated for an additional 3 weeks before sample collection (*n* = 4 animals per time point).

### Tail Amputation and Sample Collection

Mourning geckos have the natural ability to autotomize their tails and exhibit several adaptations that limit pain, hemorrhage, and tissue damage including fracture planes, decreased innervation, and arterial sphincters (Woodland, 1920; Moffat and Bellairs, 1964).

Regenerated tails were harvested at predetermined time points (7, 14, and 21 days). Prior to amputation, the tails were wiped three times with alcohol wipes to remove oils in the surface that may interfere with the fixation process. Regenerated tails were removed with a sterile #10 scalpel blade by cutting 3 mm proximally to the original amputation site with the intention to include tail stump tissues in the histology sample to allow for visualization of the boundary between original and regenerated tissues. The animals were then returned to their cages and allowed to recover. Tissue samples were then fixed overnight in 4% paraformaldehyde (Electron Microscopy Sciences).

### Immunohistochemistry

Following fixation, the samples were washed with PBS (Life Technologies), decalcified for 4 days in Versenate EDTA solution (American Master Tech). Processed samples were then taken through a sucrose gradient (10, 20, 30%), embedded in OCT compound (Tissue-Tek), sectioned (16 µm thick) on a cryotome (Leica), and mounted on glass slides (*n* = 4 section per sample). Antigen retrieval was performed with 1 mg/ml chondroitinase (Sigma-Aldrich) and 5 mg/ml hyaluronidase (Sigma-Aldrich) for 30 min at 37°C. Nonspecific binding was suppressed with 1% horse serum (Vector Labs) in PBS for 45 min. Slides were then washed with 0.1% Triton X-100/TBS, blocked in 1% BSA, incubated with primary antibodies against collagen type II (Col2) (Abcam), myosin heavy chain (MHC) (Developmental Studies Hybridoma Bank), and/or proliferating cell nuclear antigen (PCNA) (Abcam) overnight at 4°C, and incubated with fluorescently labeled secondary antibodies (Invitrogen) for 1 h at room temperature. Samples were counterstained with DAPI (Invitrogen) and imaged with an Olympus CKX41 microscope outfitted with a Leica DFC 3200 camera.

## Results

### Cartilage Cells Contribute to Blastema Formation

**Figure 1:**
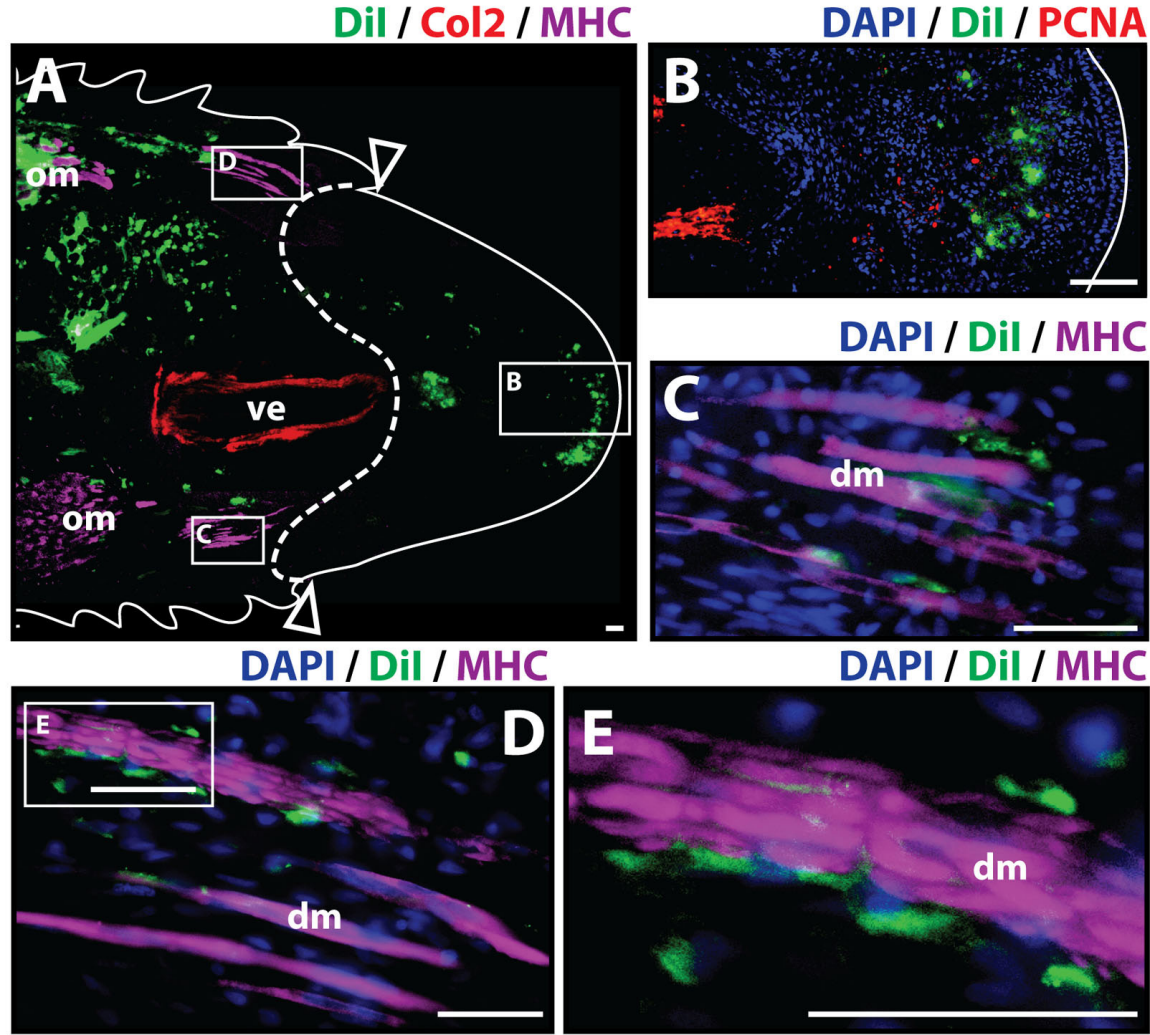
Cartilage cells contribute to the blastema. Dil-labeled (green) cartilage cells were injected into original tails and visualized histologically 7 days post-amputation. **(A)** Longitudinal tissue sections of tail stump. Tissue section containing original tissues (left of dotted line) and regenerated tissues (right of dotted line) were immunolabeled with antibodies against Collagen type II (Col2—cartilage—red) and myosin heavy chain (MHC—muscle—purple). Dil-labeled cells (green) are visualized at the original injection site in the tail stump (left of dotted line) and contributing to the blastema in the subapical space at the distal end (inset). **(B)** Higher magnification of inset in panel (A) showing the presence of Dil-labeled cartilage cells at the site of blastema formation. **(C,D)** Higher magnification of insets in panel (A) showing association of Dil-labeled cells and degenerating muscle. **(E)** Higher magnification of inset in panel **(D)**. Nuclei are stained with DAPI (blue). b, blastema; dm, degenerated muscle; om, original muscle; ve, vertebra. Bar = 75 µm.

To analyze the contribution of cartilage cells to the blastema and regenerated tissues, cartilage cells were pre-labeled *in vitro* with the fluorescent tracer Dil and injected into original tails. Two important requirements for this procedure were the verification that cartilage cell cultures were free of muscle cells prior to Dil labeling and retention of Col2 marker while *in vitro* culture to verify the differentiated state of chondrocytes throughout the duration of this process (Figure S1 in Supplementary Material). Following cell engraftment, tails were amputated at injection sites. Histologic examination of tail stumps 7 days post-amputation allowed for visualization of Dil-labeled cartilage cell distribution during the early stages of the regenerative process as blastema formation has been reported to occur as early as 1 week post-amputation (McLean and Vickaryous, 2011) (Figure 1A). Identification of original vertebral and skeletal muscle tissues within the tail stump was achieved by immunolabeling of Col2+ (red) and MHC+ (purple) cells, respectively. At 7 days post-amputation, Dil-labeled cartilage cells (green) were visualized at three different locations with the majority of cells remaining at the original injection site and smaller fractions of cells migrating to the subapical space in between the regenerated spinal cord and the AEC (Figure 1B), and adjacently to degenerating muscle (Figures 1C–E) (see Figure S2 in Supplementary Material for immunolabeling and vehicle control samples). Blastemal cells typically aggregate in the subapical space, therefore suggesting that cartilage cells contribute to the blastema.

### Cartilage Cells Contribute to Cartilage and Muscle Formation during Regeneration

**Figure 2:**
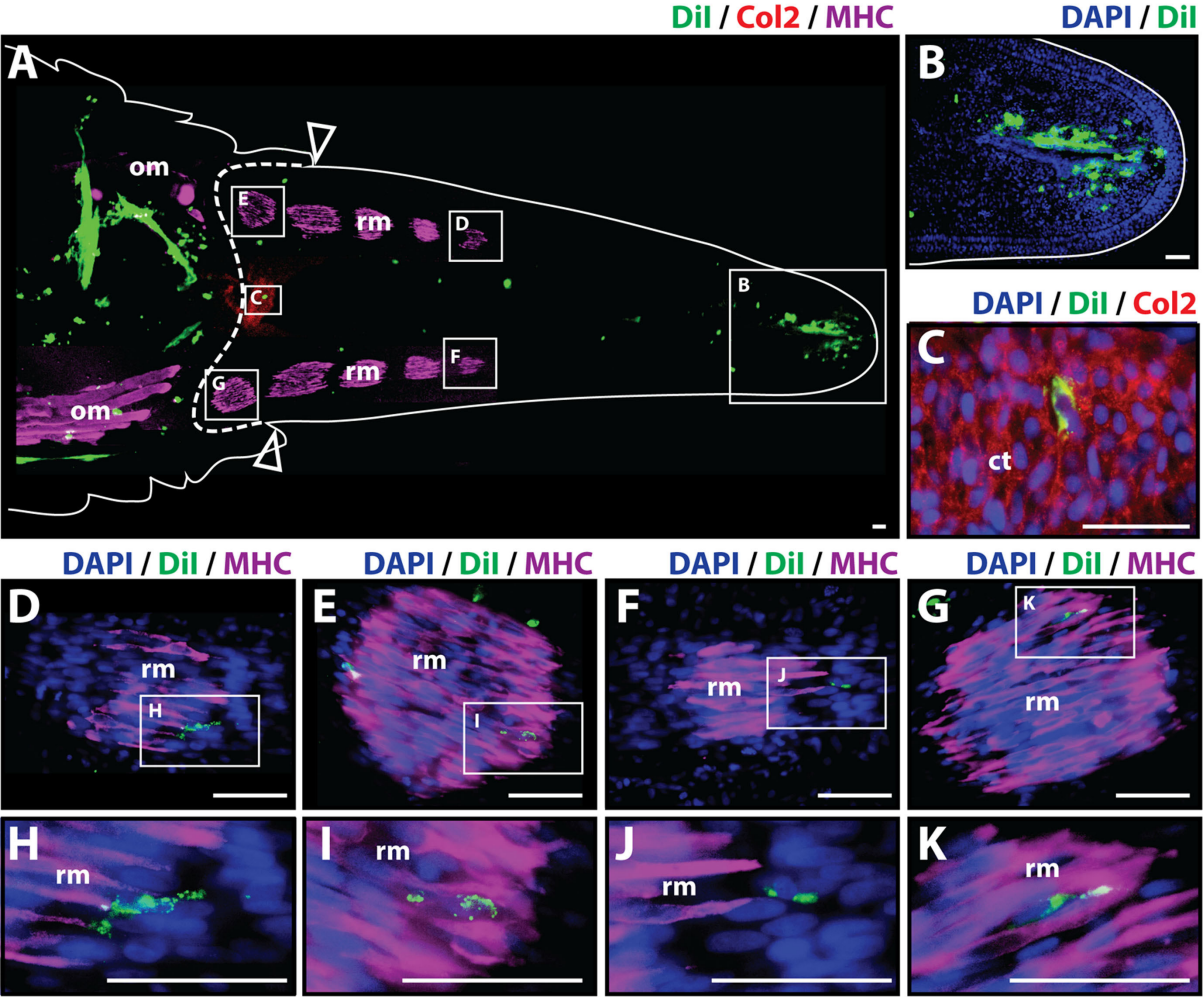
Cartilage cells contribute to cartilage and muscle formation. Dil-labeled (green) cartilage cells were injected into original tails and visualized histologically 14 days post-amputation. **(A)** Longitudinal tissue section of regenerated tail. Tissue section includes original tissues (left of dotted line) and regenerated tissues (right of dotted line). Sections were immunolabeled with antibodies against Collagen type II (Col2—cartilage—red) and myosin heavy chain (MHC—muscle—purple). Dil-labeled cells (green) are visualized at the original injection site in the tail stump (left of dotted line) and subapical space at the distal end (inset B). **(B)** Higher magnification of inset in panel (A) showing the presence of Dil-labeled cartilage cells in the subapical space. **(C)** Higher magnification of inset in panel (A) showing the colocalization of Dil-labeled cells and Col2+ staining (cartilage). **(D–G)** Higher magnification of insets in panel (A) showing colocalization of MHC+ staining (muscle) and Dil-labeled cartilage cells. **(H–K)** Higher magnification of insets of panel **(D)** through panel **(G)**. Nuclei are stained with DAPI (blue). b, blastema; om, original muscle; ct, cartilage tube; rm, regenerated muscle. Bar = 75 µm.

To analyze whether cartilage cells produce differentiated cartilage and/or muscle tissues during the regenerative process, Dil-labeled cartilage cells were injected into original tails, and the regenerated tissues were evaluated histologically at 14 and 21 days after amputation. At 14 days post-amputation the regenerated tail was about 0.5 cm long. The regenerated tail contained both a small segment of the early cartilage tube at its proximal end and islands of mature skeletal muscle scattered throughout its length as shown by Col2+ and MHC+ staining, respectively (Figure 2A). Dil-labeled cartilage cells (green) were identified in multiple locations throughout the stump and regenerated tail with the majority of cells still localizing at the original injection site in the tail stump. A more prominent contribution of Dil-labeled cells to the subapical space population was observed at 14 days in comparison to samples obtained at the 7-day time point (Figures 2A,B). Dil-labeled cells were also observed to colocalize with Col2+ cells (Figures 2A,C) and with MHC+ skeletal muscle in different segments and at various intervals throughout the regenerated tail (Figures 2A,D–K), suggesting that cartilage cells have the ability to mobilize beyond the blastema during the regenerative process and in fact, contribute to the regeneration of both cartilage and skeletal muscle tissues (see Figure S3 in Supplementary Material for immunolabeling and vehicle control samples).

**Figure 3:**
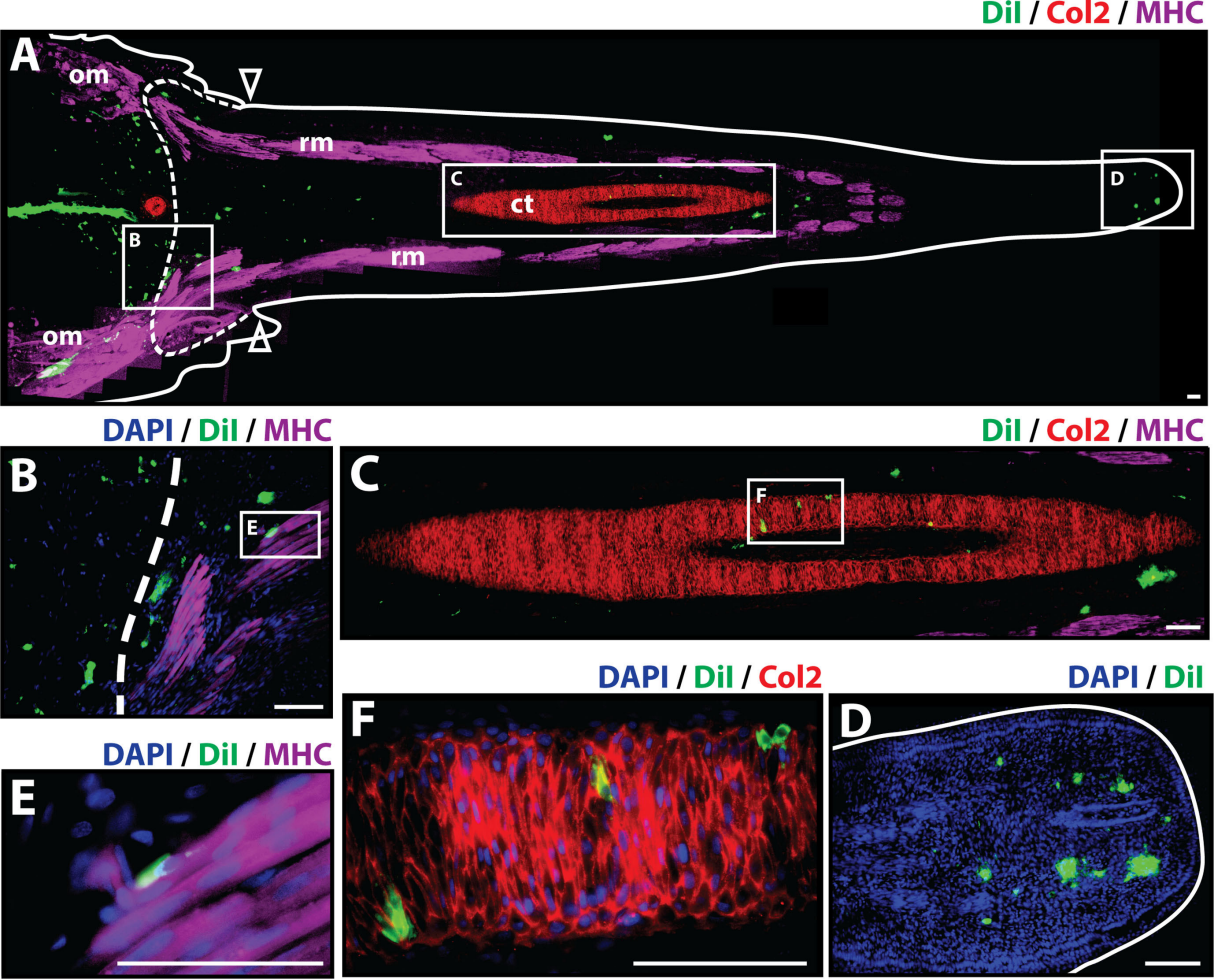
Cartilage cells contribute to cartilage and muscle formation. Dil-labeled (green) cartilage cells were injected into original tails and visualized histologically 21 days post-amputation. **(A)** Longitudinal tissue section of regenerated tail. Tissue section includes original tissues (left of dotted line) and regenerated tissues (right of dotted line). Sections were immunolabeled with antibodies against Collagen type II (Col2—cartilage—red) and myosin heavy chain (MHC—muscle—purple). Dil-labeled cells (green) are visualized at the original injection site in the tail stump (left of dotted line), colocalized with MHC+ staining (inset B), colocalizing with Col2 staining in the cartilage tube (inset C), and in subapical space at the distal end (inset D). **(B)** Higher magnification of inset B in panel (A) showing colocalization of MHC+ staining (muscle) and Dil-labeled cartilage cells. **(C)** Higher magnification of inset C in panel (A) showing colocalization of Col2+ staining (cartilage) and Dil-labeled cartilage cells. **(D)** Higher magnification of inset D in panel (A) showing Dil-labeled cartilage cells in the subapical space. **(E)** Higher magnification of inset E in panel (B) showing colocalization of MHC+ staining (muscle) and Dil-labeled cartilage cells. **(F)** Higher magnification of inset F in panel (C) showing the colocalization of Dil-labeled cells and Col2+ staining (cartilage). Nuclei are stained with DAPI (blue). b, blastema; om, original muscle; ct, cartilage tube; rm, regenerated muscle. Bar = 75 µm.

At 21 days post-amputation (Figure 3), the regenerated tail measures around 1.3 cm and a mature hollow cartilage tube can be observed along its entire length of the tail accompanied by well-organized skeletal muscle in the periphery (Figure 3A). The most prominent presence of Dil-labeled cells at 21 days post-amputation remains at the injection site. A smaller fraction of Dil-labeled cells was visible in the subapical space (Figures 3A,D), and individual cells were observed to colocalize with Col2+ staining in the cartilage tube (Figures 3C,F) and MHC+ staining in regenerated muscle (Figures 3B,E) (see Figure S4 in Supplementary Material for immunolabeling and vehicle control samples). Taken together, these observations suggest that at 21 days post-amputation, cartilage cells contribute to regenerated cartilage and skeletal muscle tissues while remaining at the subapical space as a possible reservoir for these tissues as the regenerated tail grows.

### Muscle Cells Contribute to Cartilage and Muscle Formation

**Figure 4:**
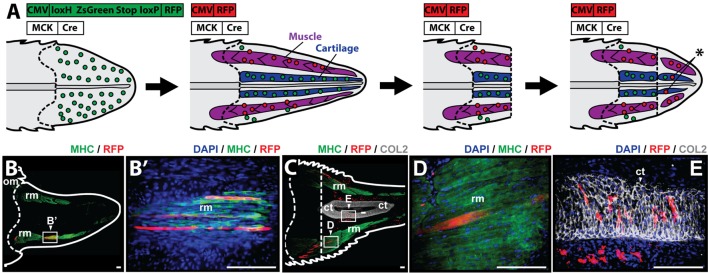
Muscle cells contribute to cartilage and muscle formation. **(A)** Experimental scheme for tracing muscle cells during lizard tail regeneration. Tail blastemas are co-transfected with muscle creatine kinase (MCK) promoter-driven Cre expression reporters and CreStoplight constructs. Activation of MCK-Cre causes mature muscle cells to switch from green-to-red fluorescence. Regenerated tails are re-amputated and regenerate, and red fluorescent cells that have incorporated into non-muscle tissues indicate reporter cells that have switched lineages (asterisk). Muscle tissue is colored purple, and cartilage is colored blue. Dashed lines mark amputation planes. **(B)** Two weeks after injection and electrophoresis, red fluorescent protein (RFP) expression colocalizes with MHC+ staining confirming confinement to myocyte lineage. **(C)** Tails are re-amputated (dotted line), and allowed to regenerate for an additional 21 days. RFP containing cells colocalize with panels **(C,D)** MHC+ muscle cells and **(C,E)** Col2+ cartilage cells. Nuclei are stained with DAPI (blue). b, blastema; om, original muscle; ct, cartilage tube; rm, regenerated muscle. Bar = 75 µm.

To study whether muscle cells contribute to differentiated cartilage and/or muscle tail tissues during the regenerative process, it was necessary to selectively label myocytes during regeneration. Myocyte lineage tracing was achieved through the use of MCK promoter-driven Cre recombinase in conjunction with a Cre-responsive green-to-red fluorescence shift construct (CreStoplight) (Figure 4A). Following injection of plasmids into tail blastemas and electroporation, tails were allowed to regenerate for 2 weeks and red fluorescent protein (RFP) expression was confirmed to be confined to the myocyte lineage by colocalization with MHC+ immunolabeling prior to re-amputation (Figure 4B). After re-amputation, lizard tails were allowed to regenerate for an additional 21 days before sample collection and immunolabeling. Histologic examination demonstrated colocalization of RFP and MHC+ cells (Figures 4C,D) as well as colocalization of RFP and Col2+ cells (Figures 4C,E) (see Figure S5 in Supplementary Material for higher magnification lineage tracing images). These findings suggest that differentiated muscle cells contribute to both regenerated cartilage and skeletal muscle tissues.

## Discussion

The present study confirms that during the process of tail regeneration, differentiated cartilage cells contribute to muscle regeneration and reciprocally, differentiated muscle cells contribute to cartilage regeneration in the mourning gecko. These findings suggest that dedifferentiation and/or transdifferentiation may be at least partially responsible for the regenerative outcome observed in this species.

The cellular origin of blastemal cells in organisms capable of epimorphic regeneration has been a topic of great interest and debate for decades (Slack, 2006). In various protostome and deuterostome organisms, both stem cells and dedifferentiated cell populations have been shown to contribute to blastemal formation. For example, in platyhelminthes and acoels, blastemas seem to form exclusively from stem cells (Bely and Nyberg, 2010), whereas in amphibian species, both mechanisms seem to contribute to this process. In a recent study, Kragl et al. (2009) used an integrated GFP transgene to track the major limb tissues during limb regeneration in the axolotl. This study showed that each major tissue produces progenitor cells with restricted potential within their embryonic layer: Dermis was able to produce cartilage and tendons, but not muscle or Schwann cells; muscle cells were able to regenerate muscle but not cartilage; cartilage was able to produce tendons and dermis, but not muscle; and in turn, Schwann cells were found to be restricted to nerve tracts. This study concluded that limb blastema cells do not switch between embryonic germ layers, although they do maintain some fate flexibility, and therefore, differentiated tissues do not necessarily have to completely dedifferentiate into a pluripotent state during limb regeneration.

The differentiation ranges during tail regeneration appear to be somewhat more promiscuous. For example, both muscle cells and GFAP+ ependymal cells contribute to cartilage cells during salamander tail regeneration (Echeverri et al., 2001; Echeverri and Tanaka, 2002). Findings in this study in the regenerated tail of the mourning gecko partially corroborate these observations. This study shows that muscle cells contribute to regenerated cartilage and that cartilage cells can form muscle during lizard tail regrowth. Thus, findings concerning regenerating tails are in direct contrast to the hard lineage restrictions reported during limb regeneration and suggest that different rules apply to limb versus tail regeneration in terms of cell differentiation potential. These differences may reflect the embryonic differences in the cell sources that make up limb vs. tail buds. For example, unlike limb buds, tail bud mesenchyme give rise to skeletal, muscular, and ectodermal lineages (Griffith et al., 1992). Future work will study the limits of lineage flexibility during tail regeneration by investigating the degree of crossing between mesodermal and ectodermal lineages.

The concept of cellular dedifferentiation is not new. Contribution from dedifferentiated cells to the regenerative process was first described by Elizabeth D. Hay based on electron microscopy studies (Hay, 1959). The concept was further confirmed *via* lineage tracing experiments using triploid axolotl donor tissue implanted into a diploid host (Namenwirth, 1974) and *via* implantation of labeled myotubes that subsequently formed multiple cell types after regeneration (Lo et al., 1993; Kumar et al., 2000). Other cell types such as Schwan cells have also been suggested to undergo dedifferentiation and metaplasia during regeneration (Wallace, 1972). However, as previously mentioned, dedifferentiation is not the only mechanism by which blastema can form in urodeles. A more recent study by Sandoval-Guzmán et al. (2014) showed that there seems to be heterogeneity with respect to the origin of blastemal cells even within closely related species. In this study, two salamander species were investigated: *Notophthalmus viridescens* (newts) and *Ambystoma mexicanum* (axolotl). This study found that myofiber dedifferentiation was an important part for limb regeneration in the newt but not in the axolotl. In the newt, myofiber fragmentation gives rise to proliferating, PAX7− mononuclear cells in the blastema that subsequently give rise to the muscle in the new limb, whereas in the axolotl, myofibers do not give rise to proliferating cells, nor do they contribute to newly regenerated muscle. Instead, resident PAX7+ cells appear to be responsible for the muscle regenerative activity in this species. The heterogeneity with respect to blastemal cell origin among closely related species may further explain the discrepancies between our findings in the mourning gecko and other regenerative organisms.

Besides heterogeneity with respect to blastemal cell origin, there are other important differences between epimorphic regeneration in amphibians and reptiles. While newts and salamanders have the ability to faithfully regenerate their limbs and tail as near perfect replicas of the original tissues (Stocum and Cameron, 2011)—and therefore are an appealing regenerative model, reptiles have a more restricted regenerative potential with only a limited number of lizard species being capable of regenerating an imperfect copy of the original tail (Bellairs and Bryant, 1985; Alibardi, 2010; Fisher et al., 2012). However, newts and salamanders are anamniote organisms and therefore do not entirely replicate the developmental processes seen in mammals. As reptiles, on the other hand, lizards share many features with other amniote organisms including similar embryonic development, thyroid-hormone-dependent transition from a two-layered periderm to cornified epithelium, absence of metamorphosis, and the presence of amnion, chorion, and allantois membranes around the embryo. Hence, reptiles more closely recapitulate many of the developmental processes seen in mammals, and as such, are a valuable tool for the study of tissue regeneration and repair and the clinical translation of these findings.

The diverse origin of cell populations that contribute to the regenerated tissues may have important implications for the regenerative process. While the proximal region of the cartilage tube undergoes ossification, the distal region resists ossification and remains indefinitely as an intact cartilage-based structure (Lozito and Tuan, 2015, 2016). This difference may be explained by the heterogeneity of the cells that contribute to the cartilage tube. Both blastemal cells and progenitor cells within the perichondrium and periosteum have been shown to contribute to CT formation (Arai et al., 2002; Yoshimura et al., 2007; Lozito and Tuan, 2015), but fate mapping studies have shown that while the ossified portion of the CT is derived from periosteal progenitor cells in response to bone morphogenic property and Indian hedgehog signaling, the cartilaginous region that resists ossification is derived from blastemal cells responding to Shh signals from the spinal cord (Lozito and Tuan, 2015). Hence, cellular origin may play a key role in the determination of cell fate in lizard tail regeneration.

Most adult mammals have very limited regenerative potential, as the default healing response after significant injury typically leads to non-functional tissue deposition with scar tissue formation. In mammals, true regeneration is restricted to a few tissues including the bone marrow, the endometrium, and to a certain extent, the epidermis. As an avascular tissue, cartilage is particularly unsuitable for regeneration (Hunter, 1995), and skeletal muscle does not have the ability regenerate after volumetric muscle loss (Pollot and Corona, 2016). Interestingly, certain species such as the MRL (Clark et al., 1998) mouse and Spiny mouse (Seifert et al., 2012) have been observed to present features of blastema-based epimorphic regeneration, and hence, understanding the mechanisms or cartilage and muscle regeneration in other amniote animals such as the mourning gecko is particularly important.

The present study has a number of limitations. First, due to difficulty obtaining a significant yield, cartilage cells for Dil labeling and tracing after tail stump injection were isolated from regenerated cartilage tubes, not from original cartilage tissues *per se*, and therefore, these finding and conclusions may not apply to original cartilage cells. Second, the presence of Dil-labeled cells in the blastema was confirmed *via* localization of Dil-labeled cells to the blastema site, not by confirmed co-expression of progenitor cell markers due to lack of antibodies against these markers in this species. Likewise, the contribution of both cartilage and muscle cells to differentiated cartilage and muscle tissues was confirmed *via* colocalization of Dil and RFP with Col2+ staining in cartilage cells and MHC+ staining in muscle cells, respectively, and was not exhaustively investigated in other tissues. Finally, we were unable to use the same experimental approach to investigate both chondrocyte and myocyte contributions. Specifically, all tested aggrecan promoter-driven constructs proved unsatisfactory for specifically labeling lizard cartilage tube cells. Thus, it was necessary to utilize Dil labeling of isolated chondrocytes, as opposed to electroporation with lineage-specific plasmids as it was done with myocytes, to trace cartilage contributions to regenerated tail tissues.

Maintenance of Col2+ staining by the embryonic growth plate-derived chondrocytes throughout the *in vitro* culture phase of the experiments confirmed the sustained differentiated state of these cells prior to injection—as differentiated chondrocytes from other species, including mammals, have been known to dedifferentiate in culture (Barbero et al., 2003; Tallheden et al., 2003), and even differentiate into myoblastic cells (de la Fuente et al., 2004). Hence, these results suggests that differentiated cartilage and muscle tissues may contribute to blastema formation and both regenerated lineages, but it does not address the question of whether this contribution occurs *via* dedifferentiation or *via* transdifferentiation nor does it examine the contribution of existing stem cell populations to regenerated tissues and therefore whether dedifferentiation/transdifferentiation is absolutely necessary for regeneration or merely contributes to this process remains to be addressed.

## Summary

In this study, we use the fluorescent tracer Dil and a creatine kinase promoter-driven Cre recombinase in conjunction with the Cre-responsive green-to-red fluorescence shift construct to trace the fate and differentiation potential of cartilage and muscles cells during tail regeneration in the mourning gecko. Our findings indicate that differentiated cartilage cells contribute to muscle tissues and reciprocally, differentiated muscle cells contribute to cartilage tissues during tail regeneration. These findings suggest that dedifferentiation and/or transdifferentiation are at least partially responsible for the regenerative outcome observed in this species.

## Ethics Statement

All procedures were approved by and performed according to the guidelines of the Institutional Animal Care and Use Committee at the University of Pittsburgh (Protocol Number 15114947).

## Author Contributions

RL, WW, BW, and TL: experimental design, data gathering/interpretation, and writing.

## Conflict of Interest Statement

This research was conducted in the absence of any commercial or financial relationships that could be construed as a potential conflict of interest.
