# Supplementary material for: Cartilage and Muscle Cell Fate and Origins during Lizard Tail Regeneration
Source: Front Bioeng Biotechnol. 2017 Nov 2;5:70. doi: 10.3389/fbioe.2017.00070 (PMC5673626; doi:10.3389/fbioe.2017.00070)
Supplement: Supplementary file 1 [file Image_1.PDF]

Supplementary Materials

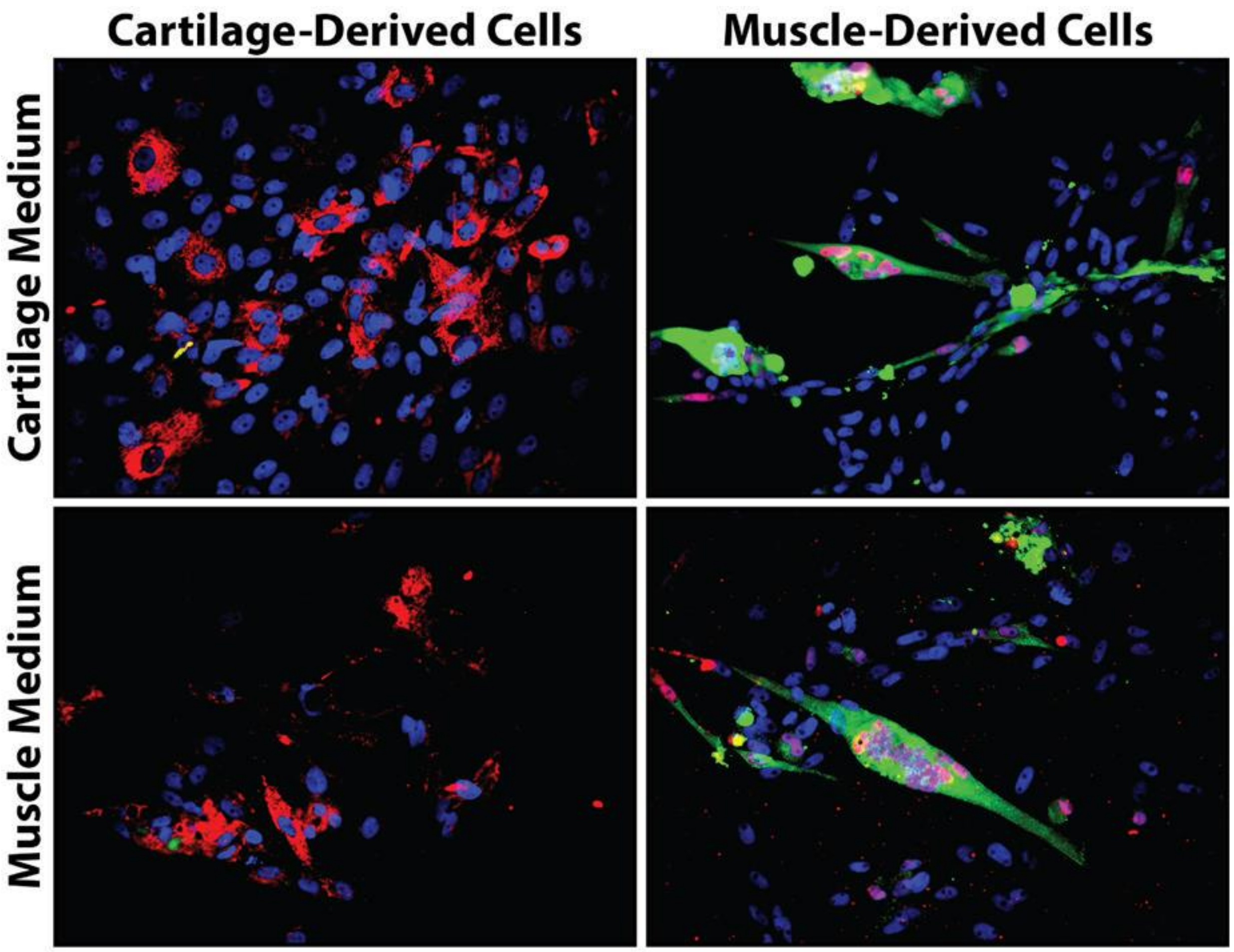

Fig. S1: Lizard cartilage cell validation. Lizard cells isolated from regenerated tail cartilage tubes and original tail muscle were cultured in either cartilage medium or muscle medium and stained for Col2 (red) and MHC (green). Nuclei were visualized with DAPI (blue). Cartilage cells exhibited Col2, but not MHC, expression regardless of media condition indicating their differentiated cartilage state throughout the duration of in-vitro culture. Similarly, muscle cells exhibited MHC expression only. These studies validated that cartilage cell cultures were free of muscle cells prior to labeling and implantation into lizard tails. Bar = 100 µm.

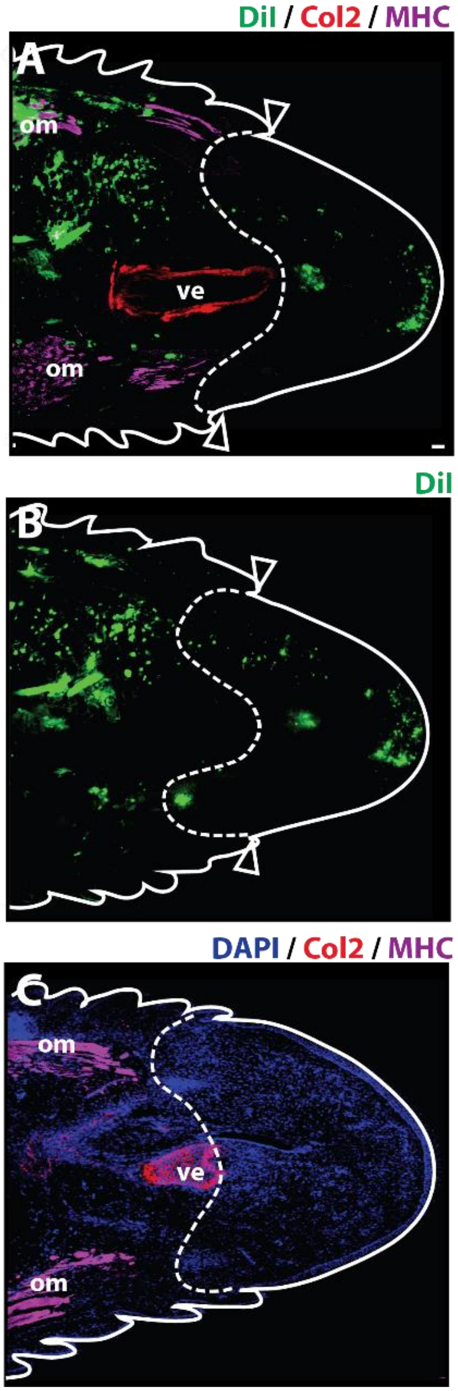

Fig S2: Negative controls for Figure 1. (A) Longitudinal tissue sections of experimental tails pre-injected with DiI-labeled (green) cartilage cells 7 days after amputation. Tails were immunolabeled with antibodies against Collagen 2 (Col2 – Cartilage - Red) and myosin heavy chain (MHC – Muscle – Purple). **(B)** Immunolabeling negative control of tail were pre-injected with DiI-labeled cells and processed identically to experimental tails expect for the omission of primary antibodies during immunolabeling. **(C)** Vehicle control tails were pre-injected with PBS instead of DiI-labeled cells. Nuclei are stained with DAPI (blue). b, blastema; dm, degenerated muscle; om, original muscle; ve, vertebra. Bar = 75 µm.

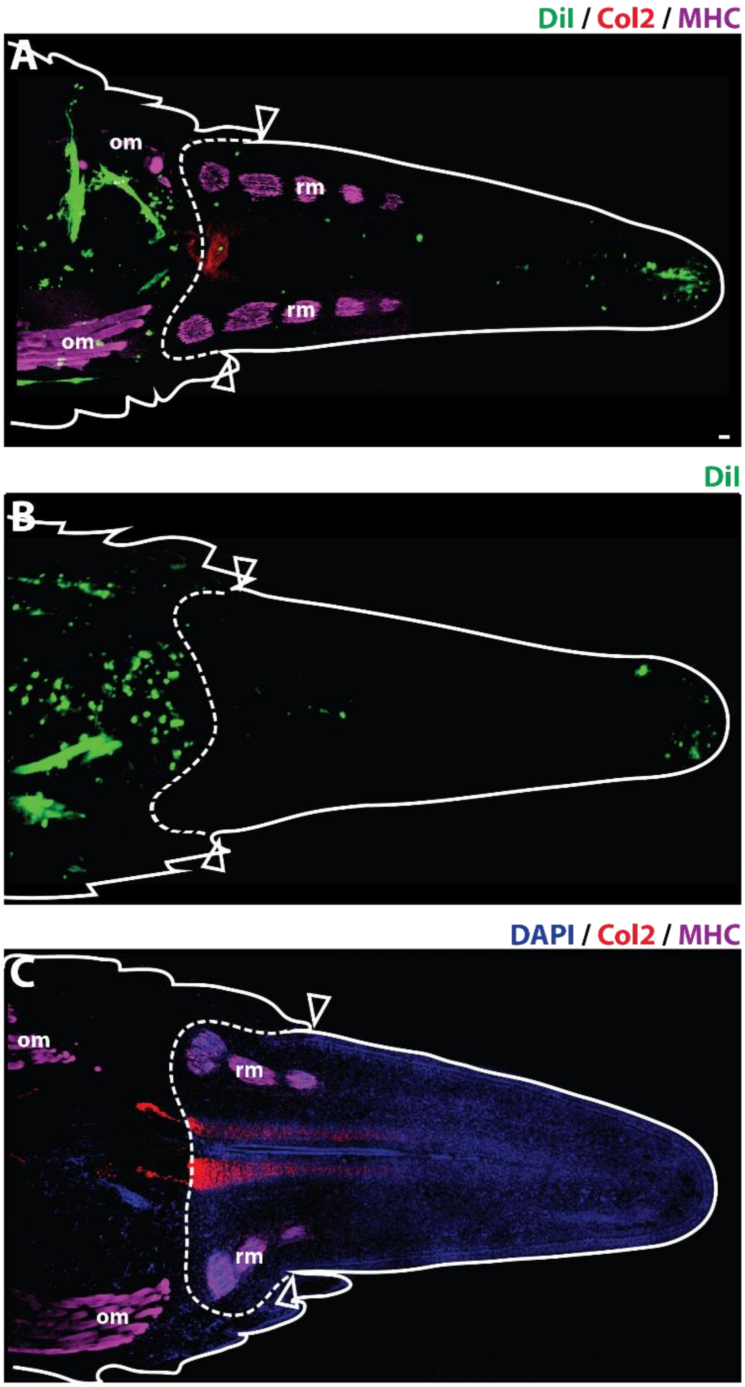

Fig S3: Negative controls for Figure 2. (A) Longitudinal tissue sections of experimental tails pre-injected with DiI-labeled (green) cartilage cells 14 days after amputation. Tails were immunolabeled with antibodies against Collagen 2 (Col2 – Cartilage - Red) and myosin heavy chain (MHC – Muscle – Purple). **(B)** Immunolabeling negative control of tail were pre-injected with DiI-labeled cells and processed identically to experimental tails expect for the omission of primary antibodies during immunolabeling. **(C)** Vehicle control tails were pre-injected with PBS instead of DiI-labeled cells. Nuclei are stained with DAPI (blue). ct, cartilage tube; om, original muscle; rm, regenerated muscle. Bar = 75 µm.

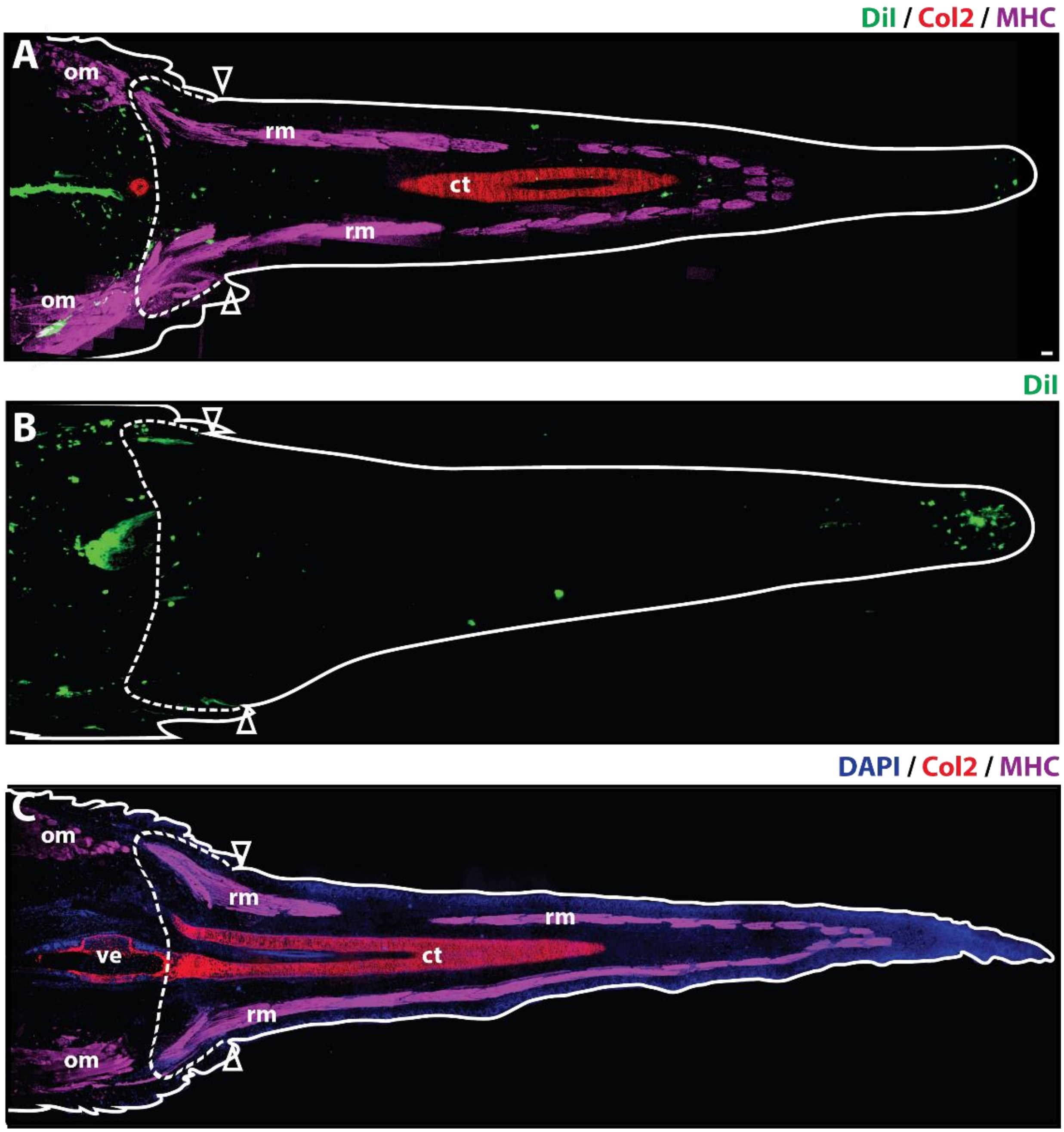

Fig S4: Negative controls for Figure 3. (A) Longitudinal tissue sections of experimental tails pre-injected with DiI-labeled (green) cartilage cells 21 days after amputation. Tails were immunolabeled with antibodies against Collagen 2 (Col2 – Cartilage - Red) and myosin heavy chain (MHC – Muscle – Purple). **(B)** Immunolabeling negative control of tail were pre-injected with DiI-labeled cells and processed identically to experimental tails expect for the omission of primary antibodies during immunolabeling. **(C)** Vehicle control tails were pre-injected with PBS instead of DiI-labeled cells. Nuclei are stained with DAPI (blue). ct, cartilage tube; om, original muscle; rm, regenerated muscle; ve, vertebra. Bar = 75 µm.

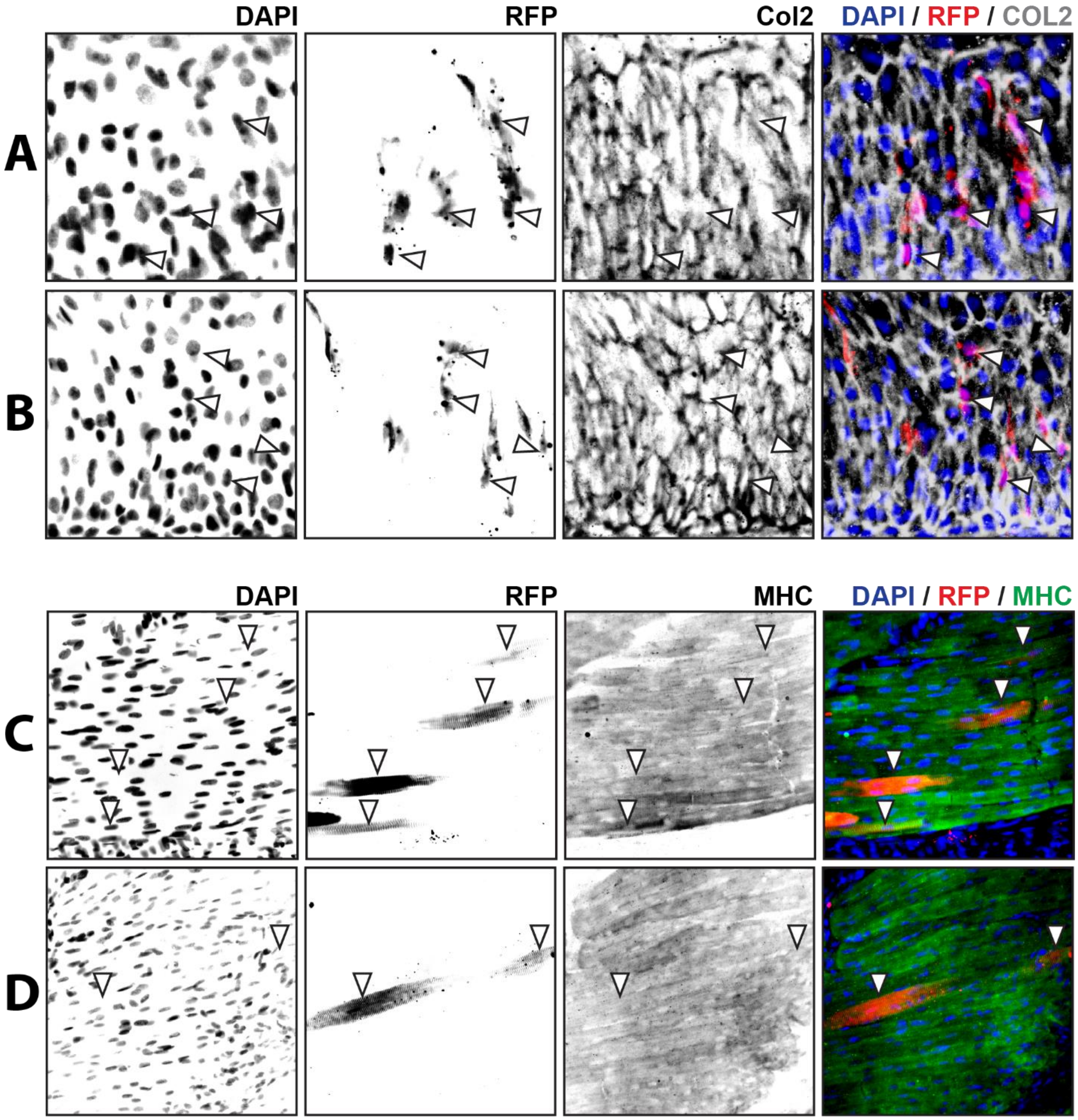

Fig S5: Validation of contribution by mature muscle cells to regenerated lizard tail cartilage. (A,B) Higher magnification views of regenerated cartilage regions showing single fluorescence channels of DAPI, RFP, and Col2 immunostaining, as well as merged images containing all three signals. Empty arrow heads identify chondrocytes labeled with RFP, indicating cells that originated as muscle cells of the original tail and became cartilage cells during tail regeneration. **(C,D)** Higher magnification views of regenerated muscle regions showing individual channels of DAPI, RFP, and MHC immunostaining, as well as merged images. Arrow heads denote muscle fibers labeled with RFP, indicating muscle tissue that originated from muscle cells of the original tail.
